# Bilateral anterior sacral meningoceles in pregnancy without sacral anomaly: a case report of a rare clinical entity

**DOI:** 10.1007/s00404-025-08294-y

**Published:** 2026-01-11

**Authors:** Carla Oelgeschläger, C. Berg, B. Grüttner, T. Groten, E. C. Weber

**Affiliations:** 1 Division of Prenatal Medicine, Gynecological Ultrasound and Fetal Surgery, Department of Obstetrics, University Hospital Cologne, Kerpener Strasse 34, 50931 Cologne, Germany; 2 Department of Obstetrics, University Hospital Cologne, Cologne, Germany

**Keywords:** Anterior sacral meningocele, ASM, Pregnancy, Bilateral, Intrapelvic, MRI, Ultrasound, Case report

## Abstract

This report describes a 32-year-old primigravida diagnosed with bilateral anterior sacral meningoceles without bony defect of the sacrum during pregnancy. The patient remained asymptomatic throughout the pregnancy, with regular monitoring via transvaginal ultrasound and MRI. An elective cesarean section was planned at 38 weeks. However, the patient presented in obstructed labor at 42 weeks and underwent an emergency cesarean section, resulting in the birth of a healthy infant. This case is unique as it involves bilateral anterior meningoceles without sacral anomalies. Anterior sacral meningoceles are rare findings in pregnancy. Anterior sacral meningoceles are either congenital with bony defect of the sacrum or acquired lesions due to connective tissue disorders characterized by the herniation of the meninges through the sacral foramina. In pregnancy, these lesions pose unique challenges due to potential complications such as rupture, infection, or obstructed labor. Management strategies vary, and individualized approaches with close monitoring and patient counseling are crucial in determining the appropriate mode and timing of delivery.

## Take-home message

**Table:** 

Anterior sacral meningoceles in pregnancy are rare and often associated with sacral anomalies. This is a unique case of bilateral anterior sacral meningoceles without associated bony defect in pregnancy highlighting its clinical presentation and its potentially different obstetric implication and management.	

## Case report

**Fig. 1:**
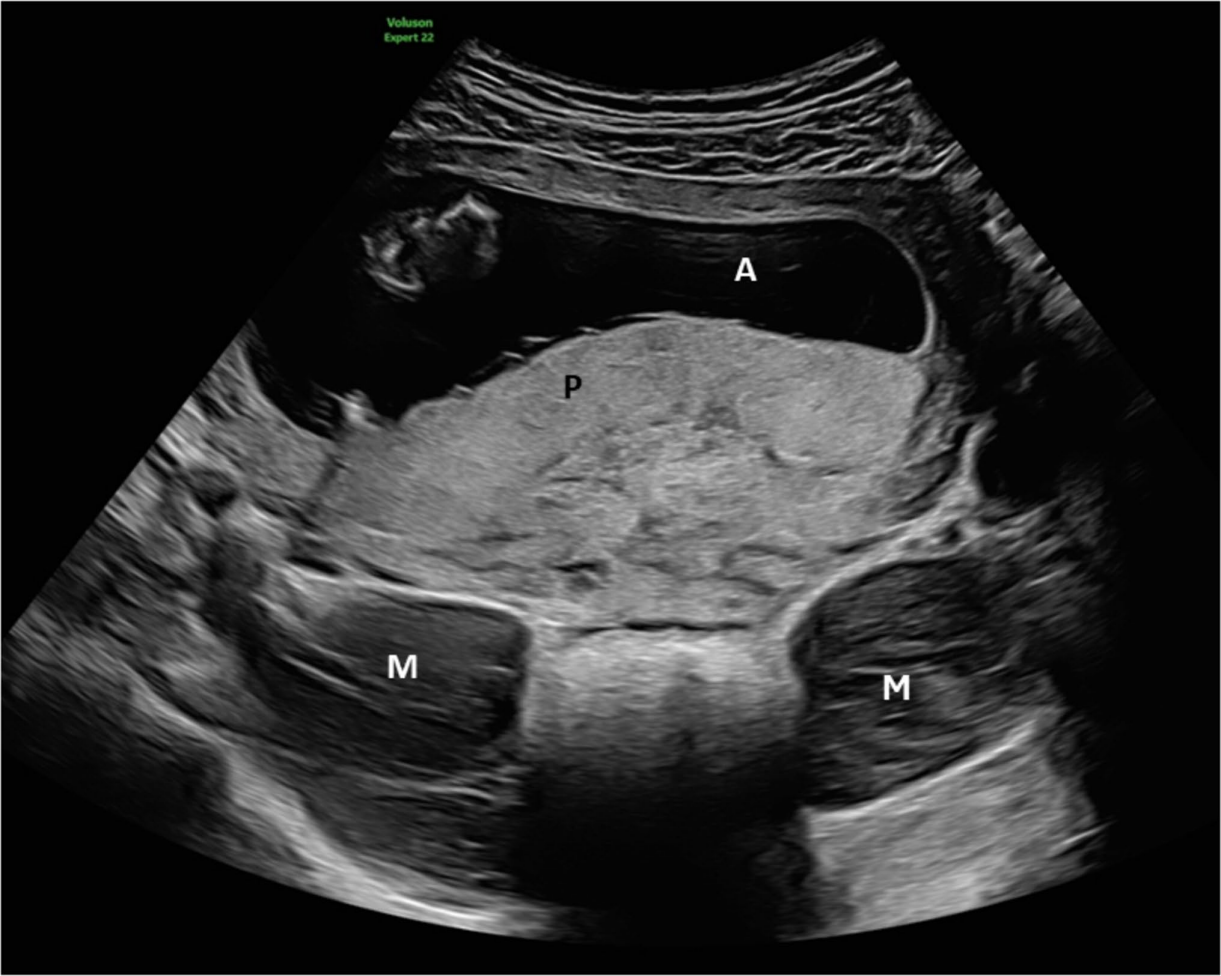
Abdominal scan at 13 weeks’ gestation showing bilateral sacral meningoceles posterior to the uterus, presenting as round shaped, cystic masses with echogenic internal structures M = meningocele, P = placenta, A = amniotic cavity

**Fig. 2:**
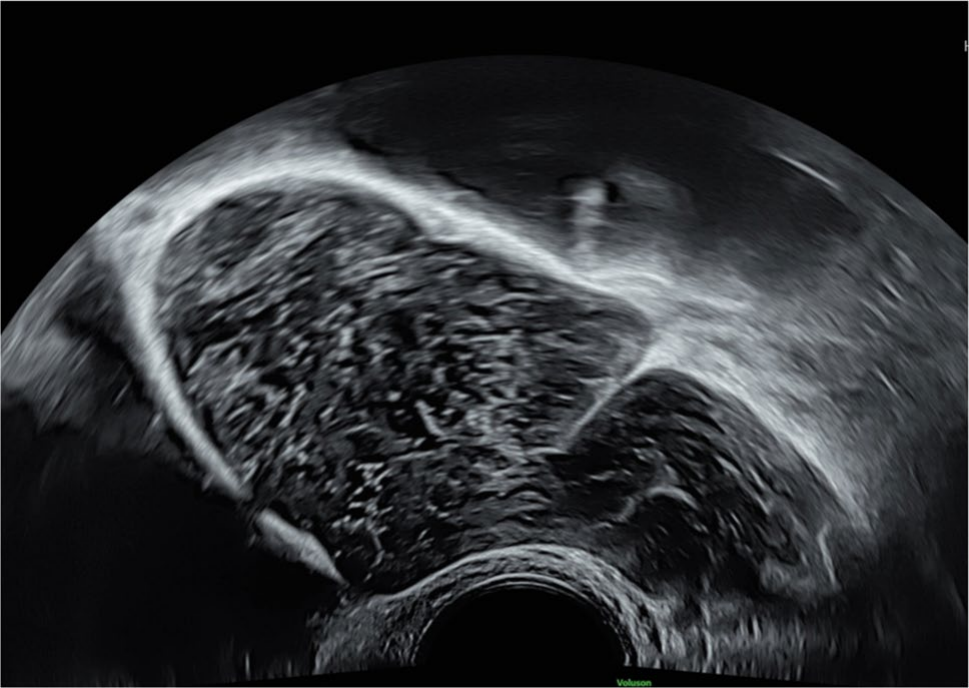
Vaginal scan at 22 weeks’ gestation showing sacral meningocele

**Fig. 3:**
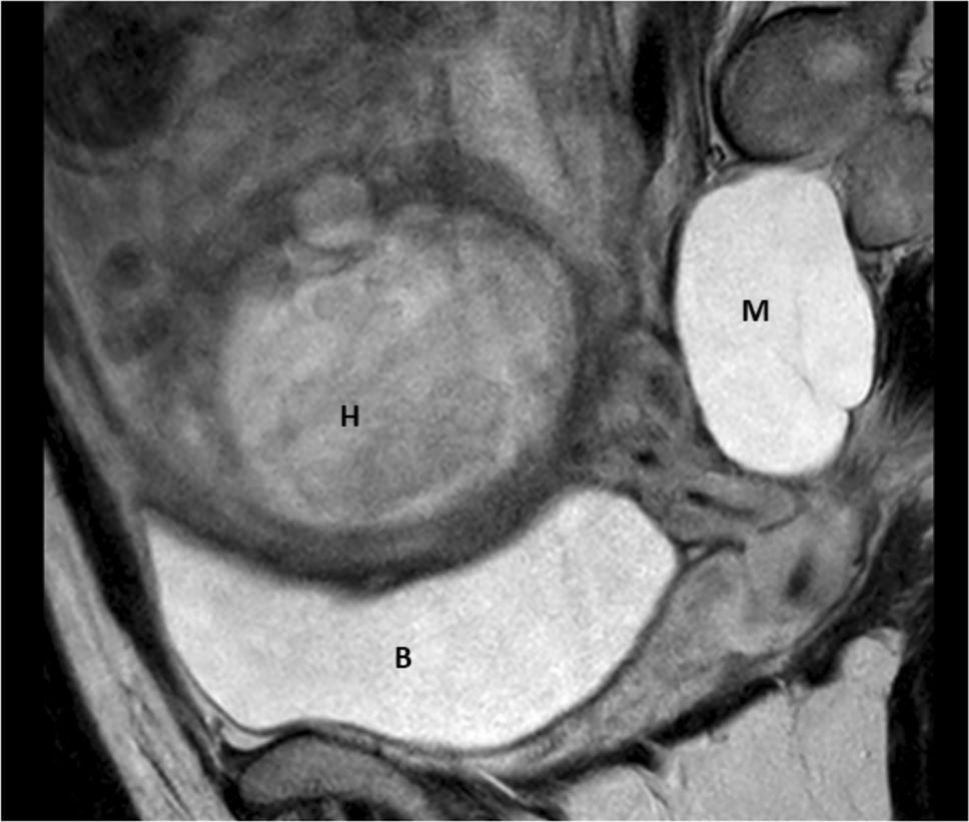
Sagittal T2-weighted MRI image at 30 weeks’ gestation showing right-sided meningocele with only slight indentation by fetal head and uterus M = meningocele, H = fetal head, B = bladder

A 32-year-old primigravida was referred for first-trimester screening at 13 weeks of gestation (Fig. 1). Six months prior, she had undergone MRI due to a suspected ovarian cyst, which revealed bilateral intrapelvic sacral meningoceles measuring 7.5 cm on the right and 5 cm on the left, extending through the S1 neuroforamina, without associated sacral bony defects. The patient was asymptomatic and declined further genetic evaluation despite a marfanoid habitus. Throughout the pregnancy, the patient remained asymptomatic. Serial transvaginal ultrasounds showed stable meningocele size (Fig. 2). At 30 weeks, MRI demonstrated slight indentation of the meningoceles by the growing uterus but no signs of compression (Fig. 3). An elective cesarean section was planned at 38 weeks; however, the patient was lost to follow-up. At 42 weeks, the patient presented in obstructed labor at a birthing center and underwent an emergency cesarean section under general anesthesia. A healthy male infant weighing 3650 g was delivered. Postpartum recovery was uneventful, and both mother and child were discharged on day four.

## Discussion

Anterior sacral meningoceles (ASM) in pregnancy are rare, with less than 20 cases reported in the literature [1–4]. ASM are either congenital with bony defect of the sacrum or acquired lesions due to connective tissue disorders or characterized by the herniation of the meninges through the sacral foramina or could be isolated [2, 5, 6]. To our best knowledge, this is the first report of bilateral ASM in pregnancy without sacral anomalies. In pregnancies with ASM, the growing uterus can pose potential complications such as meningocele compression resulting in increased intracranial pressure with headaches, meningocele rupture with subsequent meningitis as the most dangerous complication, and labor obstruction [1]. In our case, the pregnancy course was uneventful, perhaps because bilateral meningoceles could better evade to the side than median ones and are therefore less compressed by the growing uterus and less symptomatic or it could be a matter of size, as the bilateral meningoceles of our case are smaller than some of the reported cases [3].

In past decades, high maternal mortality was reported, largely due to complications during labor or postpartum rupture [1, 7, 8]. Therefore, some authors advocate for early cesarean delivery (e.g., at 35–36 weeks) to avoid potential complications [1, 2, 4]. However, with advancements in imaging (especially MRI) and prenatal care (especially widespread use of ultrasound), individualized risk assessments and delivery management plans can be developed. Our case supports a more individualized approach: with close monitoring and stable findings, term delivery may be appropriate in asymptomatic women. Moreover, this case highlights the complexity of patient autonomy in high-risk pregnancies. Despite extensive counseling and a detailed plan at a tertiary center, the patient chose to deliver naturally at a birthing center. Although emergency intervention ensured a good outcome, this scenario underscores the need to align medical safety with patient preferences—potentially by offering vaginal delivery in a controlled hospital setting, when risk is acceptable.

Bilateral ASM without sacral anomaly are exceedingly rare in pregnancy. With appropriate monitoring and individualized management, term delivery can be safely achieved. Literature on this topic is limited, and this case highlights the need for further research to establish optimal management strategies.

## Data Availability

No datasets were generated or analysed during the current study.
